# Debate: Is ACE screening doing more harm than good? A consideration of screening versus child health surveillance

**DOI:** 10.1111/camh.70123

**Published:** 2026-09-28

**Authors:** Helen Minnis

**Affiliations:** ^1^ Centre for Developmental Adversity and Resilience, School of Health and Wellbeing University of Glasgow Glasgow UK

**Keywords:** ACEs, screening, child health surveillance

## Abstract

The ACEs questionnaire has been used in population epidemiology for decades and is now recommended, by some authors, for screening purposes. However, screening programmes bring both harms and benefits and are different from population epidemiology and clinical enquiry. In this paper, I use a modern modification of Wilson and Jungner's screening programme criteria to consider whether a screening programme for ACEs would be advisable. I conclude that a screening programme for ACEs would not be appropriate and might be harmful. However, routine child health surveillance, with close collaboration across agencies including health, education and social work, is essential to ensure that ACEs, and potential harms arising from ACEs, are avoided wherever possible. A composite case example from Scotland, a country with a well‐developed child health surveillance system, is provided to illustrate how such a system can support children who have experienced ACEs and their families.

**Case Example:** *This is a composite of real cases (details changed to protect identity) from Scotland, a wealthy country with a reasonably well‐functioning child health surveillance system and policy that supports multi‐agency preventative work to prevent ACE‐related harms (Getting it Right for Every Child, GIRFEC*  https://www.gov.scot/policies/girfec/ *)*.

A woman phones social work concerned that her neighbour, Ms Davis, has been seen in the neighbourhood drunk in charge of her 20‐month‐old daughter Amy. A social worker visits and Ms Davis tells her she has occasionally been binge drinking because of the stress of her recent separation and lack of support with childcare. Social work convenes a multi‐agency meeting involving the health visitor, GP and nursery (where Amy attends 2 days per week). Nursery staff say Ms Davis seems like a caring parent, but they thought they smelled alcohol on her breath once or twice. The GP recalls Amy crawling on his lap at the surgery and the Health Visitor recalls similar indiscriminate behaviour during a home visit. Both are concerned that Amy's language development is delayed.

A referral is made to the local Infant Mental Health team who notice Amy's indiscriminate behaviour, delayed language and a strained mother–infant relationship. They explain to Ms Davis that Amy's indiscriminate behaviours are likely related to the impact of her drinking on her ability to care for her daughter and that they will have to discuss this with social work. Ms Davis is very shocked, admits her drinking has been more than occasional, and the social worker helps her arrange support for her alcohol misuse. Ms Davis begins video interaction guidance with the IMH team to support her in getting her relationship with Amy back on track. The social work team continue surveillance of the family and Amy is given an additional day in nursery.

Ms Davis successfully stops drinking, with the support of her mother who takes on some additional care of Amy. Three years later, Amy has caught up in her language development and is about to start school.

*All screening programmes do harm; some do good as well, and, of these, some do more good than harm at reasonable cost*.

Gray, Patnick, & Blanks (2008, p 480)

The ACEs questionnaire has been beneficial in demonstrating the population‐level associations between childhood adversity and negative health outcomes, and for demonstrating the high prevalence of childhood adversities, and variations in prevalence, worldwide (Gordon & Felitti, 2023).

However, population epidemiology and screening are two different things. The originators of the ACEs questionnaire advocate ACE screening in ‘every medical encounter’ (Gordon & Felitti, 2023)—while cautioning that, for children, ‘the causal diagnosis may have challenging reporting and legal consequences’ (Gordon & Felitti, 2023). Therin lies the rub: the *causal* relationship between ACEs and negative health outcomes is poorly understood.

Firstly, despite increasing population‐level risk of negative health outcomes, at an individual level, ACEs often do not result in poor health. In adults, the modal response to a potentially traumatic event is resilience—defined as ‘stable psychological and physical health from before to after the potentially traumatic event’ (Galatzer‐Levy, Huang, & Bonanno, 2018). Resilience across all domains is less common after childhood adversities such as maltreatment, but complete recovery can still occur (Topitzes, Mersky, Dezen, & Reynolds, 2013). Secondly, ACEs research is subject to important confounders. People who have experienced ACEs are more likely to be neurodivergent, with largely heritable diagnoses such as ADHD or Autism (Dinkler et al., 2017) being independently associated with negative health outcomes (Catala‐Lopez et al., 2022). Thirdly, the prevalence of ACEs, and their associations with psychopathology, vary across the world (Pace, Muzi, Rogier, Meinero, & Marcenaro, 2022), demonstrating the importance of culture and context. Finally, in the absence of good family support services, a child might be taken into care when serious harm has not occurred and when family support might have prevented harm.

By cautioning against ACEs screening, I am not suggesting we should not *ask* our patients about ACEs, where appropriate. Adult self‐reports of childhood maltreatment are reliable and associated with psychopathology (Coleman, Al‐Jaber, Aponsu, Stahl, & Danese, 2026), so clinicians need the courage and training to explore this with our patients. However, sensitive clinical enquiry and screening are two different things.

So, what is screening? In 1968, Wilson and Jungner launched a set of 10 criteria to ensure screening programme benefits outweigh the harms. Table S1 summarises a recent updating of these criteria (Dobrow, Hagens, Chafe, Sullivan, & Rabeneck, 2018). Taking these criteria into consideration, I do not advocate for ACEs screening.

I do, however, advocate for high‐quality child health surveillance, that is, a structured programme of routine preventive checks, physical assessments and developmental screenings for infants and young children such as is available in many wealthy countries. In other words, it is important for countries to have systems in which children experiencing harm from ACEs can be identified and supported. In the UK, health visitors provide crucial surveillance to identify childhood health problems and harms as early as possible, and our social care system is also available for more intensive child‐welfare surveillance of children living in high‐risk families. Where such programmes work well, they share many of the attributes of the ‘program/system principles’ described in Table S1: close liaison and collaboration between health, education and social work services and a careful case‐by‐case weighing up of the costs and benefits of intervening early to prevent ACEs and avoid associated harms.

However, some wealthy countries, including the US, do not have universal child health surveillance, and it is perhaps unsurprising that many calls for ACEs screening come from the US. Research is ongoing to try to determine the benefits, harms and costs of early‐intervention for ACEs: there is evidence that ensuring that the various components of such a complex system work well together is challenging (Crawford et al., 2025).

## Conclusion

I do not advocate for ACEs screening because it likely causes more harm than good. I do, however, advocate for all countries to develop high‐quality multi‐agency child health surveillance systems. Such surveillance should include consideration of whether children are experiencing harm resulting from ACEs so that the right support can be offered and, at a population level, governments should carefully monitor the programme quality and costs (both personal and financial) to ensure that the benefits outweigh the harms.

## Conflict of interest statement

The author declares no potential conflicts of interest.

## Funding information

No funding source supports the writing of the paper.

## Ethics statement

Ethical approval is not applicable to this debate article.

## AI statement

The Author declares that they have not used AI in any capacity that would require disclosure in accordance with Wiley's AI Guidelines, nor in any capacity that would reasonably require disclosure for editors, reviewers and readers to properly evaluate their research or manuscript. The author confirms that AI has not been used for purposes including but NOT limited to drafting and editing, to generate substantial text or restructure arguments, nor in the research methodology. The author takes full responsibility for the accuracy of this statement.

## Supporting information


**Table S1.** Principles to consider when evaluating whether a screening programme is advisable, based on consideration of the original Wilson and Jungner criteria, a systematic review and consensus process—modified with permission from Dobrow, Hagens, Chafe, Sullivan, & Rabeneck, 2018 doi:10.1503/cmaj.171154.

## Data Availability

Data sharing not applicable to this article as no datasets were generated or analysed for this debate article.

## References

[camh70123-bib-0001] Catala‐Lopez, F. , Hutton, B. , Page, M.J. , Driver, J.A. , Ridao, M. , Alonso‐Arroyo, A. , … & Tabares‐Seisdedos, R. (2022). Mortality in persons with autism spectrum disorder or attention‐deficit/hyperactivity disorder: A systematic review and meta‐analysis. JAMA Pediatrics, 176, e216401. 10.1001/jamapediatrics.2021.6401 PMC8845021 35157020

[camh70123-bib-0002] Coleman, O. , Al‐Jaber, M. , Aponsu, G. , Stahl, D. , & Danese, A. (2026). Stability of childhood maltreatment self‐reports: A systematic review and meta‐analysis. Nature Mental Health, 4, 1315–1328. 10.1038/s44220-026-00677-7 PMC13441976 42564127

[camh70123-bib-0003] Crawford, K. , Young, R. , Wilson, P. , Deidda, M. , Forde, M. , Millar, S. , … & Ougrin, D. (2025). Infant mental health services for birth and foster families of maltreated pre‐school children in foster care (BeST?): A cluster‐randomized phase 3 clinical effectiveness trial. Nature Medicine, 31, 1617–1625. 10.1038/s41591-025-03534-9 PMC12092239 40312587

[camh70123-bib-0004] Dinkler, L. , Lundstrom, S. , Gajwani, R. , Lichtenstein, P. , Gillberg, C. , & Minnis, H. (2017). Maltreatment‐associated neurodevelopmental disorders: A co‐twin control analysis. Journal of Child Psychology and Psychiatry, and Allied Disciplines, 58, 691. 10.1111/jcpp.12682 28094432

[camh70123-bib-0005] Dobrow, M.J. , Hagens, V. , Chafe, R. , Sullivan, T. , & Rabeneck, L. (2018). Consolidated principles for screening based on a systematic review and consensus process. Canadian Medical Association Journal, 190, E422–E429. 10.1503/cmaj.171154 PMC5893317 29632037

[camh70123-bib-0006] Galatzer‐Levy, I.R. , Huang, S.H. , & Bonanno, G.A. (2018). Trajectories of resilience and dysfunction following potential trauma: A review and statistical evaluation. Clinical Psychology Review, 63, 41–55. 10.1016/j.cpr.2018.05.008 29902711

[camh70123-bib-0007] Gordon, J.B. , & Felitti, V.J. (2023). The importance of screening for adverse childhood experiences (ACE) in all medical encounters. AJPM Focus, 2, 100131. 10.1016/j.focus.2023.100131 PMC10546489 37790951

[camh70123-bib-0008] Gray, J.M. , Patnick, J. , & Blanks, R. (2008). Maximising benefit and minimising harm of screening. BMJ, 336, 480–483. 10.1136/bmj.39470.643218.94 PMC2258553 18310003

[camh70123-bib-0009] Pace, C.S. , Muzi, S. , Rogier, G. , Meinero, L.L. , & Marcenaro, S. (2022). The Adverse Childhood Experiences–International Questionnaire (ACE‐IQ) in community samples around the world: A systematic review (part I). Child Abuse & Neglect, 129, 105640. 10.1016/j.chiabu.2022.105640 35662684

[camh70123-bib-0010] Topitzes, J. , Mersky, J.P. , Dezen, K.A. , & Reynolds, A.J. (2013). Adult resilience among maltreated children: A prospective investigation of main effect and mediating models. Children and Youth Services Review, 35, 937–949. 10.1016/j.childyouth.2013.03.004 PMC3640482 23645949

