# Supplementary material for: Debate: Is ACE screening doing more harm than good? A consideration of screening versus child health surveillance
Source: Child Adolesc Ment Health. 2026 Sep 28;31(4):351–3. doi: 10.1111/camh.70123 (PMC13668478; doi:10.1111/camh.70123)
Supplement: Supplementary file 1 [file CAMH-31-351-s001.docx]

**Table S1.** Principles to consider when evaluating whether a screening programme is advisable, based on consideration of the original Wilson and Jungner criteria, a systematic review and consensus process - modified with permission from Dobrow et al, 2018 doi: 10.1503/cmaj.171154

| **Domain** | **Screening principles** | **Consideration of the screening principle for ACEs** |
| --- | --- | --- |
| Disease or condition | 1. Epidemiology of the disease or condition | An ACE is not a disease or condition – and may or may not lead to a disease or condition. |
|  | 2. Natural history of disease or condition | ACEs increase disease risk but are also often associated with health. |
|  | 3. Target population for screening | We do not yet know who, in the population, will develop ACE-related diseases or conditions, or why. |
| Test/intervention principles | 4. Screening test performance characteristics | Self-report of ACEs is stable in adults but less so in children (Coleman et al., 2026), so there is a danger of *false negatives* (i.e. missing ACEs when they are/have been present) in children.  There is also a likelihood of high levels of *false positives* (i.e. determination that harm leading to diseases or conditions has taken place when it has not). |
|  | 5. Interpretation of screening test results | There is danger that an assumption of ACE-related harm could increase patient/family anxiety and hopelessness. |
|  | 6. Post-screening test options | Clinicians would need to liaise closely with social work colleagues, if ACEs are reported in children, to determine the child’s safety and risk of harm. |
| Program/system principles | 7. Screening program infrastructure | To develop an effective ACEs screening programme for children, there would need to be close liaison between health, education and social work services. |
|  | 8. Screening program coordination and integration | Close multi-agency collaboration would be required to ensure that there is the lowest possible rate of false-positives and false-negatives in ACEs detection. |
|  | 9. Screening program acceptability and ethics | Careful consideration would be needed to ensure that the harms associated with, for example, removing a child into state care, were less than the harms associated with leaving the child in a family where ACEs have occurred. |
|  | 10. Screening program benefits and harms | At a population level, close scrutiny would be required to determine the harms and benefits of detecting ACEs and of how that knowledge is acted upon. |
|  | 11. Economic evaluation of screening program | The immediate and lifespan personal and societal costs and benefits of identifying ACEs and associated harms would need to be weighed up. |
|  | 12. Screening program quality and performance management | There would need to be strong policy oversight to ensure that the various components of the program work well together in order to minimise program-related harms. |
